# The adaptive value of recombination in resolving intralocus sexual conflict by gene duplication

**DOI:** 10.1098/rspb.2024.2629

**Published:** 2025-01-22

**Authors:** Jon Alexander Harper, Edward H. Morrow

**Affiliations:** ^1^ Evolution, Behaviour and Environment Group, School of Life Sciences, John Maynard Smith Building, University of Sussex, Brighton BN1 9QG, UK; ^2^ Department of Environmental and Life Sciences, Karlstad University, Karlstad 651 88, Sweden

**Keywords:** sexual antagonism, sexual conflict, sexual dimorphism

## Abstract

Recombination plays a key role in increasing the efficacy of selection. We investigate whether recombination can also play a role in resolving adaptive conflicts at loci coding for traits shared between the sexes. Errors during recombination events resulting in gene duplications may provide a long-term evolutionary advantage if those loci also experience sexually antagonistic (SA) selection since, after duplication, sex-specific expression profiles will be free to evolve, thereby reducing the load on population fitness and resolving the conflict. The potential advantage of gene duplication may be tempered by the short-term deleterious effects on gamete and zygote survival, which may be tolerable in a species with high reproductive output but not with low reproductive output. We used datasets of candidate SA loci from *Drosophila melanogaster* and humans to test these ideas. As in humans, sexually antagonistic alleles in flies with net positive effects across the two sexes occurred at higher frequencies than alleles with net negative effects. In flies, higher recombination rates were associated with more intense levels of sexual conflict and genes with paralogues occur in regions with higher recombination rates, indicating gene duplication events are associated with a history of SA selection. Genes experiencing higher levels of conflict also showed both a higher proportion with paralogues and higher numbers of paralogues. Together, our findings reveal multiple lines of evidence for a possible route towards the resolution of an adaptive conflict via gene duplication that is facilitated by higher recombination rates.

## 1. Introduction

Differences in how natural and sexual selection act on males and females cause an adaptive conflict at genetic loci coding for traits expressed in both sexes. As a result, sexually antagonistic (SA) selection generates a population-level ‘load’ [1–3], or reduction in fitness as alleles are maintained in the population that are deleterious to either one of the two sexes. Unresolved, SA selection is therefore expected to limit the ability of males and females to evolve phenotypes with optimal fitness, but may help maintain genetic variation [4,5]. The evolution of sexual dimorphism should in theory alleviate this load as a constraint to phenotypic evolution (perhaps only partially), but it requires the evolution of additional (genetic or epigenetic) mechanisms or evolutionary steps [6–8].

Multiple mechanisms have been proposed that could resolve intralocus sexual conflict (IASC) and alleviate or eliminate the sexual dimorphism/gender load [9]. For traits where gene expression mediates the strength of sexual conflict, the evolution of sex-biased expression can be a route to resolution [10,11]. For coding regions, mechanisms of conflict resolution may be more complex, requiring multiple evolutionary steps, including the evolution of modifiers, alternative splicing, translocation to sex chromosomes, sex-specific dominance reversal, genomic imprinting [6,8,12–17] or the duplication of entire genes followed by sub-functionalization [18,19].

The resolution of IASC via gene duplication is particularly interesting since it is one of the few mechanisms that would have an immediate and significant impact on genomic architecture, as new sequences are generated from the duplication event and incorporated into the genome [7,19,20]. Gallach and Betrán [19] outlined one model for how conflict resolution via gene duplication may occur. They envisaged this process to be driven by conflict over genes that need to function across male-specific (e.g. testes) and non-male-specific tissues, thereby experiencing a kind of ‘tissue antagonism’, as well as conflict between the sexes. Selection would then favour duplication of the gene segregating for SA alleles, followed by testis-specific expression of the male-benefit allele, which may be facilitated by gene relocation [19–21]. A recent study of the tandem gene duplication *Apollo* and *Artemis* in *D. melanogaster* by VanKuren and Long [22] provides empirical support for this model, where an ancestral conflict over actin structure in gametogenesis was partially resolved by duplication and sub-functionalization. *Apollo* expression in males is required for spermatid maturation and sperm individualization while *Artemis* expression in females is required for correct egg morphology. However, deletions of *Apollo* in females or *Artemis* in males result in a 15–19% increase in reproductive output in the respective sex. This indicates that, to some extent, the sexual dimorphism/gender load partially remains, and resolution of IASC is not complete, despite the ancestral gene duplicating. This evidence illustrates that an association between the presence of SA selection at a locus and gene copy number variation can arise due to the process of gene duplication because gene duplication *per se* does not necessarily fully resolve IASC—it is instead only the first step of an evolutionary process where additional steps are required before the sexual dimorphism/gender load can be affected.

If a genetic locus experiences SA selection, what adaptive mechanisms could enhance the probability that a duplication event will occur at that locus? Duplications to particular genomic regions or single genes are broadly thought to occur due to either replicative transposition (and retrotransposition) or unequal crossing-over [23]. Both transposition and unequal crossing-over are dependent on the process of recombination. Recombination rates are known to vary across the genome in many organisms, but the adaptive significance of this variation is not fully resolved [24–26]. Recombination not only plays a role in the origin of gene duplications, it also allows selection to more easily remove deleterious rearrangements, increasing the efficacy of both purifying and positive selection, and reducing the strength of Hill–Robertson interference [27–29]. For example, Wilfert *et al.* [30] argued that recombination rates may be especially high in social Hymenoptera, as sociality strongly selects for increased genotypic diversification, which intra-chromosomal crossovers would provide. Recombination rate therefore has a direct impact on the evolutionary dynamics of chromosomal rearrangements such as gene duplications, not only by generating them but also by efficiently exposing them to selection.

There are several studies that have empirically explored the relationship between recombination rate and various measures of genomic structural variation that have accumulated through evolutionary time, with mixed results. In a study of avian models, recombination rates were found to be higher in regions containing copy number variants (CNVs) compared to regions without CNVs [31], which is consistent with the hypothesis that recombination increases the probability of gene duplication events occurring. A comparative study of angiosperms found a significant positive correlation across species for recombination rate in euchromatin and gene family size [32]. Positive relationships between recombination rate and tandemly arrayed gene density were also found in *Arabidopsis thaliana* [33] and rice [34], but there was no relationship between recombination rate and gene density in a butterfly species [35]. In *D. melanogaster*, recombination rate data from DNA sequences were subdivided into 4 levels and the number of gene duplications occurring in these 4 groups was counted [36]. The authors found a nominally U-shaped relationship, where the highest numbers of duplications occurred in regions with the lowest recombination rates and another peak in regions with the highest recombination rates. One interpretation is that in regions with very low recombination rates, any duplications that do occur persist as selection is inefficient at removing them. In regions with the highest recombination rates, deleterious duplications occur more frequently, but they are also more efficiently eliminated by selection, while beneficial duplications may accumulate. We are not aware of any studies that have examined the relationship between recombination rate and incidence of gene duplications or copy number variants in humans, but on balance there does seem to be accumulating evidence across a range of taxa that duplications are more likely to occur in regions with higher rates or crossing-over.

We hypothesize that there may be a long-term evolutionary advantage to an increase in recombination rate at loci experiencing strong IASC because it would increase the probability of gene duplication events, and therefore facilitate conflict resolution via subfunctionalization [7,19,20]. The benefits may not be large enough to outweigh the costs of direct selection against increasing recombination rate at loci with weaker IASC [37]. Through its potential to change gene copy number, recombination may have an adaptive value beyond the classical advantages of increasing the efficiency of selection, genetic diversity, and rates of adaptation (reviewed in [28]). Specifically, we predict that (i) regions with more intense sexual antagonism should coincide with higher recombination rates, and (ii) SA genes on average have a higher number of paralogues than genes not experiencing sexual antagonism, since there exists an association between extant IASC and duplicated genes that can persist over substantial periods of evolutionary time [22]. This is because duplication is only one step towards full conflict resolution. Although recombination is limited to females in Drosophila, indirect selection on recombination via males is still able to operate over the long-term, since both sexes harbour genetic variation for recombination rate, selection on recombination rate can occur via any female offspring males have. Elevations in recombination rate may, however, incur a cost, as processes such as non-allelic homologous recombination and meiotic events can result in deletions or aneuploidies [38], as well as duplications [39]. All can be deleterious for gametes, developing zygotes or adults, and can cause infertility as well as a range of other genomic disorders [39,40]. These costs may be particularly hard to bear for organisms that follow a life-history strategy of high investment in a few slow-growing offspring but may be more tolerable for species that produce large numbers of fast-growing offspring. We therefore make a more specific version of prediction (i) that, with all else being equal, the short-term costs associated with elevated recombination rate (as a hypothesized way of promoting gene duplication at SA loci) may be favoured by selection in species with high reproductive output, such as the fruit-fly, but may be disfavoured by selection in species with low reproductive output, such as humans. Therefore, in the context of conflict resolution, we predict (iii) there may be an association between the strength of sexual conflict and recombination rate in the fruit fly but not in humans. We test these predictions here, as lists of candidate SA loci are now available in both species [41,42]. We also test whether the allele frequencies at SA loci in flies are higher when the net effects of the alleles across the two sexes are more positive, as predicted by a population genetic model [43], first by looking across all loci, then by subdividing loci according to different functional categories that the genes influence.

## 2. Methods

### (a) Population genetics

Our primary dataset consisted of a list of 2372 candidate SA SNPs within 337 genes in *Drosophila melanogaster* (FDR < 0.3) identified in a recent genome-wide association study (GWAS) of SA genetic variation [42]. In addition, we used a dataset on human SA variants, taken from a recent meta-analysis of the biomedical literature [41] that identified genes with SA effects from various GWAS and candidate gene studies. The effect sizes from the *D. melanogaster* data (*z*-scores) were converted to Cohen’s *d* using the formulas in Koricheva *et al*. [44] to make them directly comparable to the published Cohen’s *d* values in humans. We note that, although these effect sizes are then directly comparable in terms of Cohen’s *d* values, this does not mean they are directly comparable proxies for fitness as the fly data are based on laboratory-based estimates of lifetime reproductive success while the human data are based on a variety of methods showing SA effects on complex traits and disease. Ruzicka *et al*. [42] used the variant effect predictor to classify the candidate SNPs into 12 functional categories [45]. We grouped the candidate SNPs into three broader functional categories: (i) missense, (ii) synonymous and (iii) regulatory variants (which included any SNPs found in 3′ UTR, 5′ UTR, downstream, upstream, intergenic, intron, non-coding transcripts, splice acceptor, splice donor and splice region variants). We reasoned that these three categories may show different compositions in terms of the relative number of valid candidates and false positives for a given genome-wide false discovery rate. For instance, missense variants may most likely include valid SA candidates as they change the amino acid in their codon and are in exons of genes. Missense variants would lead to an altered gene product or affect regulation, while synonymous variants are perhaps more likely to be neutral, but see [46] and therefore may be more likely to be dominated by false positives. It is much more difficult to predict whether the regulatory variants will be dominated by valid candidates or false positives. Although sexually antagonistic patterns of gene regulation are known to exist [47], the list of candidates is derived from allelic variation, not variation in expression level, and so have less direct effects on fitness relative to missense variants. Dividing regulatory variants further does not seem justifiable, as we currently see no distinct predictions for these other more fine-scaled categories.

The ratio of fitness effect sizes of an allele across the two sexes (calculated by dividing the beneficial effect by the deleterious effect) provides a single metric where the value indicates whether the net effect is either beneficial or detrimental (following [43]). It does not provide a direct estimate of the strength or intensity of the conflict, for that we use a different index described below. The effect size ratio will always take a negative value for SA alleles, since one effect size will be positive and one effect size will be negative. Alleles with an effect size ratio less than −1 have a beneficial effect in one sex that is greater than the deleterious effect in the opposite sex, while alleles with an effect size ratio greater than −1 have a detrimental effect in one sex that is greater than the beneficial effect in the opposite sex. Our *D. melanogaster* dataset contained only minor alleles (found in <50% of the studied population). Since effect sizes for these alleles were calculated relative to the opposite allele in every case, it is possible to calculate the effect sizes for major alleles as well, but these data are not independent of one another and investigating relationships between these groups is therefore not statistically valid.

We began by modelling how effect allele frequency varied with effect size ratio using a generalized linear model (GLM), since models predict higher allele frequencies when they have net beneficial effects [43]. We initially investigated this relationship across all data, using variant functional category as a covariate, along with the interaction between variant functional category and effect size ratio. We then stratified alleles by variant functional category, fitting weighted linear models of allele frequency as a function of effect size ratio to each category separately.

### (b) Conflict resolution

#### (i) Recombination rates

To test the hypothesis that there is a relationship between the intensity of sexual conflict and recombination rate, we needed a single measure of conflict intensity. Innocenti and Morrow [48] proposed a sexual antagonism index (SAI) based on the standardized directional selection gradients for a given trait in males and females, but transforming Cohen’s *d* values to selection gradients (beta) requires reliable estimates of standard deviation, which were not available for all data points. Instead, here, we use a modified version of this sexual antagonism index:


$$SAI=\;\frac{d_{M}d_{F}}{\sqrt{\frac{d_{M}^{2}+d_{F}^{2}}{2}}}$$

where *d_M_* and d*_F_* are the effect sizes for a particular allele in males and females, respectively. As with the original formulation of SAI, it gives a proxy measure of conflict intensity, which is mathematically superior to averaging across sexes or the sex difference or ratio in effect sizes, as it is proportional to the absolute intensity of SA effects [48]. SAI differs from the ratio of effect sizes outlined above in that it describes the magnitude of conflict experienced at a particular locus instead of the balance between beneficial and deleterious effects. For example, a SNP that confers large effects of identical magnitude but opposite in direction between the sexes would yield a strongly negative SAI, while the ratio between such effects would be net neutral.

Recombination rate data in *D. melanogaster* were obtained from Comeron *et al*. [49], where sequences from marked *D. melanogaster* genomes were used to detect crossover events. Data from their study constitute the most recent complete recombination map for *D. melanogaster* but used coordinates based on release 5 of the *D. melanogaster* genome assembly, while the Ruzicka *et al*. [42] dataset used release 6 coordinates. We therefore used the Flybase coordinates converter to match these datasets [50]. As the resolution of the *D. melanogaster* recombination map is limited, missense SNPs within each gene were pooled to calculate a mean SAI for missense SNPs in each gene experiencing sexual antagonism (i.e. harbouring at least one candidate SA SNP). Human recombination rate data were obtained from the DeCODE recombination map [51]. Investigating the relationship between SAI and recombination rate was not possible with a generalized linear modelling approach due to heteroscedasticity, and we were unable to find a suitable link function or transformation that gave satisfactory diagnostics [52]. Instead, we divided SAI data from both *D. melanogaster* and human genes into three equal-sized quantiles (tertiles): low, medium and high conflict intensity. Recombination rate across these three categories was then compared using non-parametric Mood’s median tests and any significant results were investigated further with *post hoc* pairwise median tests.

#### (ii) Gene duplications

Data on all documented *D. melanogaster* paralogue and singleton genes, including those that are not known to contain any SA loci, were obtained from Flybase [50]. For the purposes of this analysis, gene pairs with a DIOPT score of 1 or greater were considered paralogues [53] (i.e. where a particular gene pair is supported by at least one bioinformatic tool). We counted the number of paralogues each gene has and assigned genes to families of paralogues. Dividing the genes into the three categories of conflict, we tested for differences in the proportion of genes with and without paralogues with a χ^2^ test, and for differences in the number of paralogues with a Wilcoxon rank-sum test. All analyses were performed in R v. 4.1.3.

## 3. Results

### (a) Population genetics

**Figure 1.**
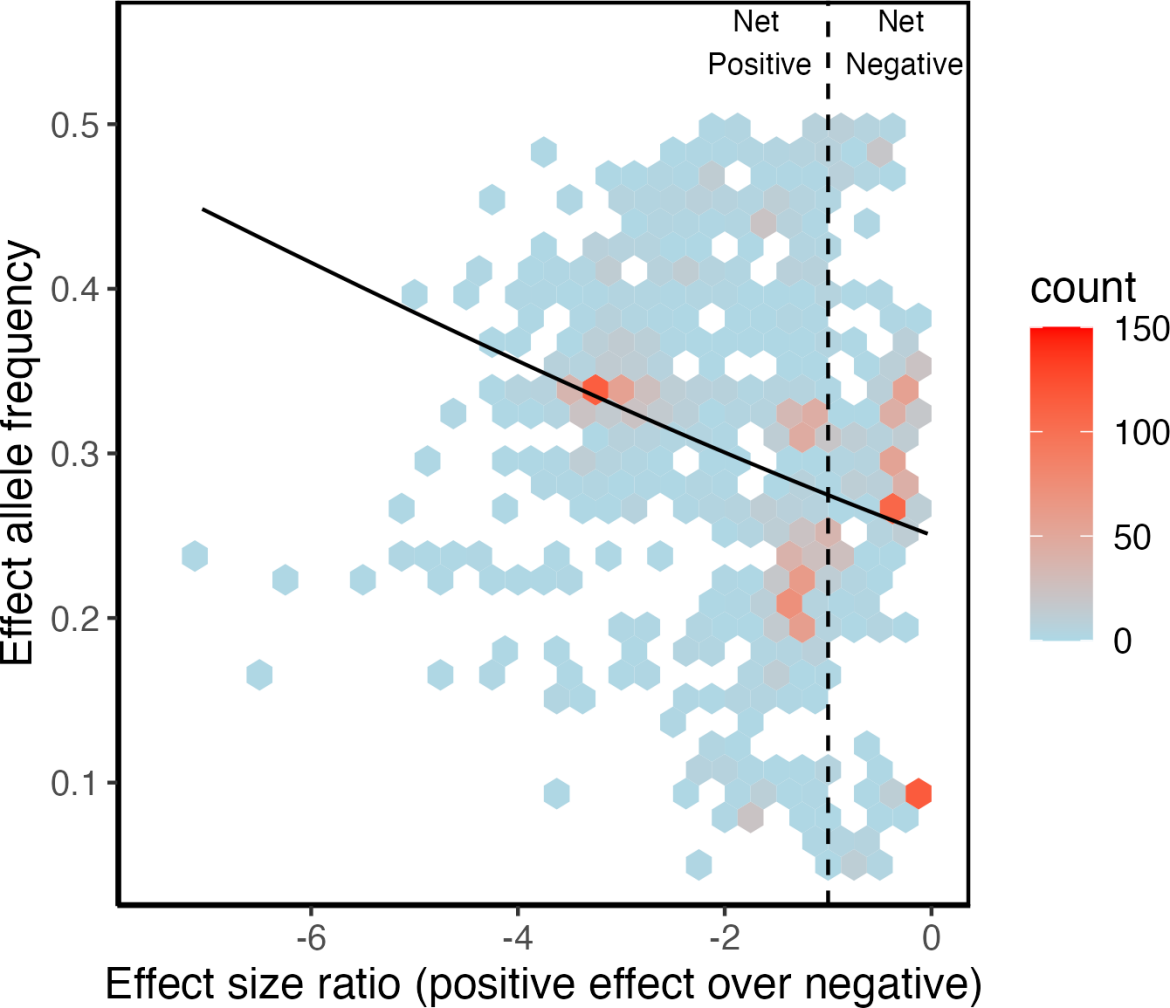
The relationship between effect allele frequency and effect size ratio in candidate SA loci. SNPs to the left of the vertical black dotted line have net beneficial effects (effect size ratio <−1) while those to the right have net deleterious effects (effect size ratio >−1). Areas with hexagons are where one or more SNPs can be found. The colour of each hexagon relates to SNP counts per hexagon (see legend). The black line represents predicted values from a linear model (see text for details).

**Figure 2.**
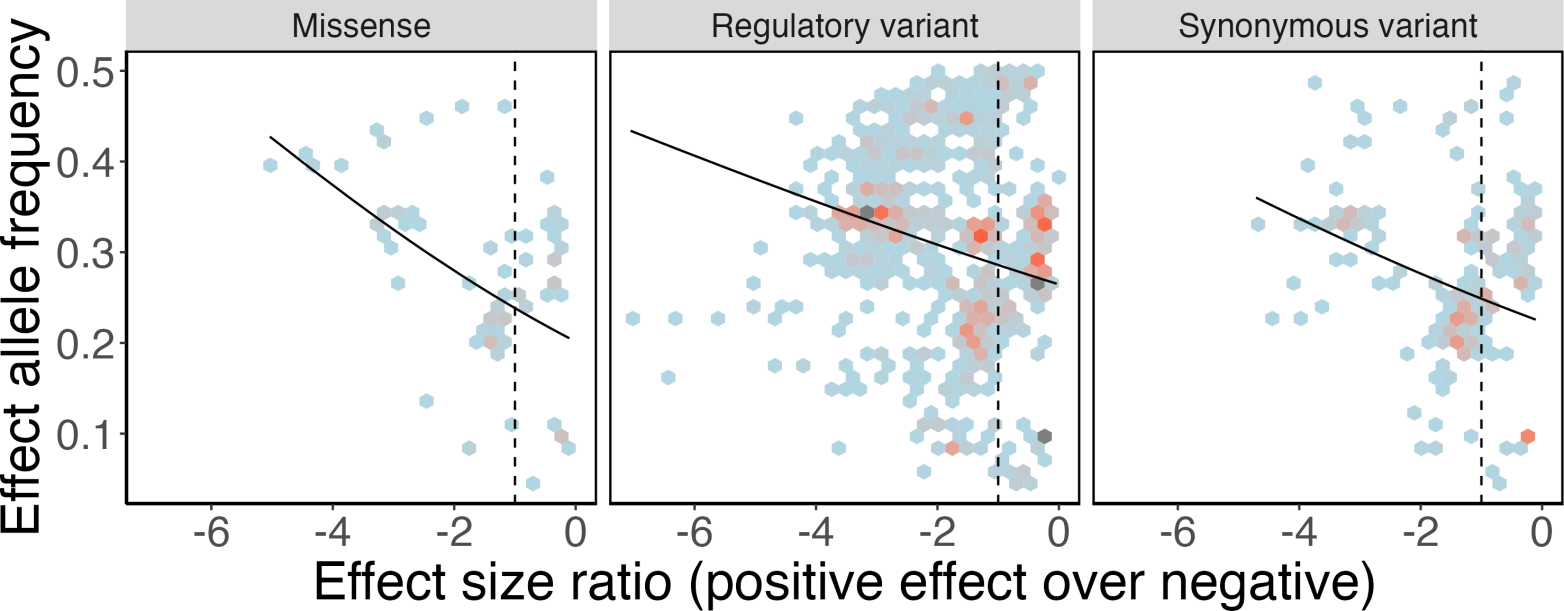
The relationship between effect allele frequency and effect size ratio across variant functional categories. Lines represent predicted values from linear models, present for the classes where significant relationships (*p* < 0.05) were detected. As shown in figure 1, the vertical black dotted line delineates net beneficial (to the left) and net deleterious effects, and coloured hexagons represent SNP density.

Across all 2372 candidate SA loci in *D. melanogaster*, we detected a negative relationship between effect size ratio and allele frequency, such that alleles with net positive effects occur at higher frequencies than alleles with net negative effects (GLM: estimate ± SE −0.126 ± 0.017, d.f. = 12 371, deviance = 24 281, *p* = 2 × 10^−16^; figure 1). This result reflects that already found across the panel of human candidate SA loci [41]. Variant functional category had a significant impact on allele frequency (deviance = 534, d.f. = 1.2, *p* = 3.01 × 10^−14^), as did the interaction between effect size ratio and variant functional category (deviance = 89.44, d.f. = 1.2, *p* = 5.35 × 10^−3^). For this reason, we investigated the effect size ratio and allele frequency relationship separately for each variant functional category. All three categories showed a significant negative relationship between effect size ratio and allele frequency (table 1; figure 2).

**Table 1.** Stratified generalized linear models. Models of how allele frequency varies with effect size ratio, stratified by variant type. Models are GLM weighted by inverse variance. RSE: residual standard error.

functional category	slope	deviance	*p*‐value	RSE
missense	−0.215	323.83	9.18 × 10^–13^	2.52
synonymous	−0.143	356.22	7.06 × 10^–13^	2.63
regulatory	−0.107	1145.69	4.17 × 10^–29^	3.02

### (b) Conflict resolution

#### (i) Recombination rates

**Figure 3.**
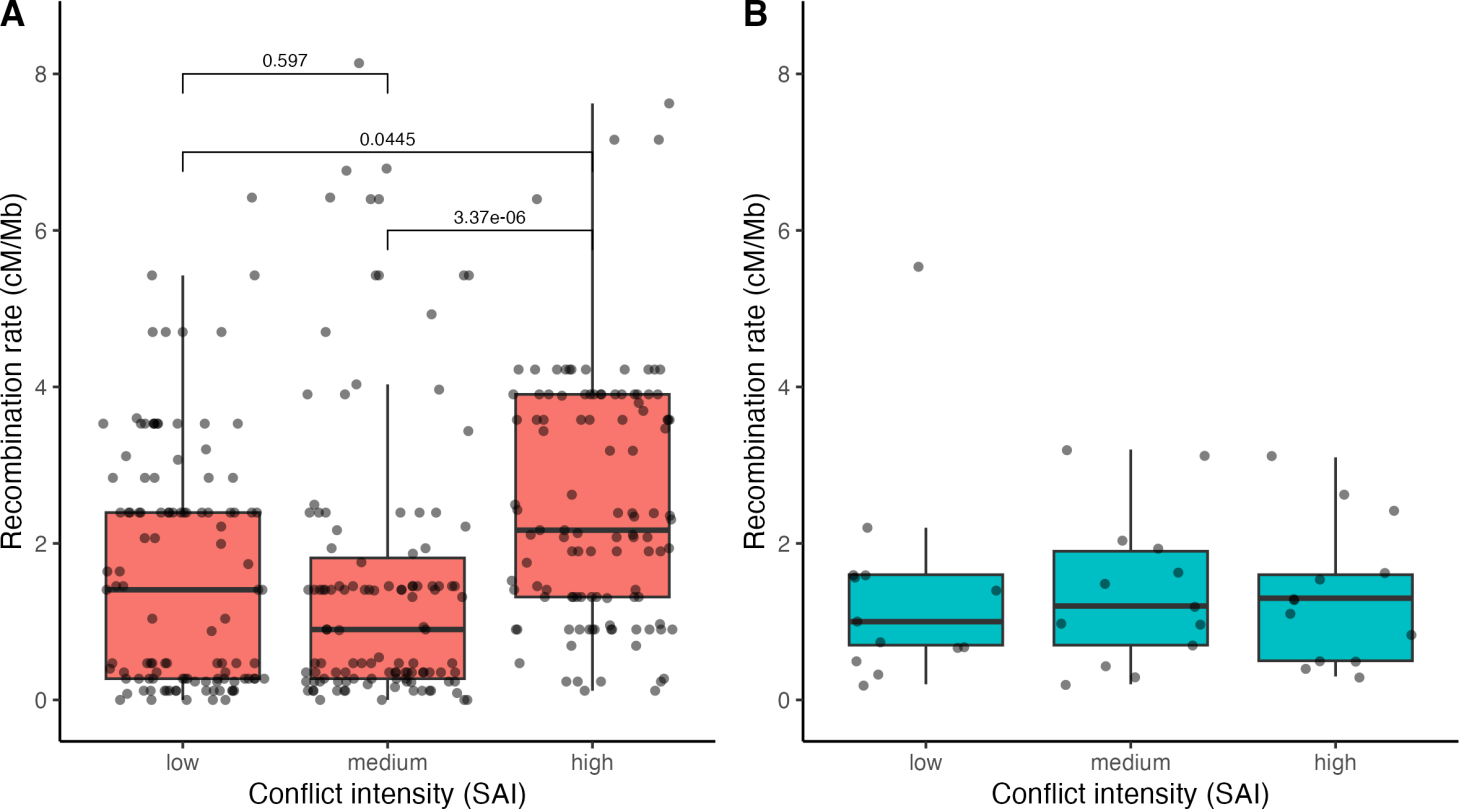
Recombination rates of SA genes are higher in the presence of strong conflict in *D. melanogaster* but not in humans. Recombination rate as a function of variation in sexual antagonism index (SAI). SAI categories were determined by the tertile of each gene. (A) *D. melanogaster*. (B) Human.

We began by grouping all the *D. melanogaster* candidate SA variants together for median testing. This test revealed differences in recombination rate across the three categories of conflict intensity (Brown–Mood median test, χ^2^ = 21.68, d.f. = 2, *p* = 1.96 × 10^−6^; figure 3A). No significant differences in the variation in recombination rate between the three categories were detected (Levene’s test, F = 0.187, *p* = 0.8296).

In contrast, SA variants in humans failed to show any statistically significant differences across the three categories of conflict intensity in terms of median (Brown–Mood median test, χ^2^ = 0.2, d.f. = 2, *p* = 0.9048) or variance in recombination rate (Levene’s test, F = 0.1063, d.f. = 2, *p* = 0.8994; figure 3B). However, with the measures used here, in humans SAI also appeared to be lower (Brown–Mood median test, Z = −6.250, *p* = 4.11 × 10^−10^) and more variable (Levene’s test, F = 34.2, d.f. = 1, *p* = 1.11 × 10^−8^) than in *D. melanogaster* (but see §2; electronic supplementary material, figure S1).

**Figure 4.**
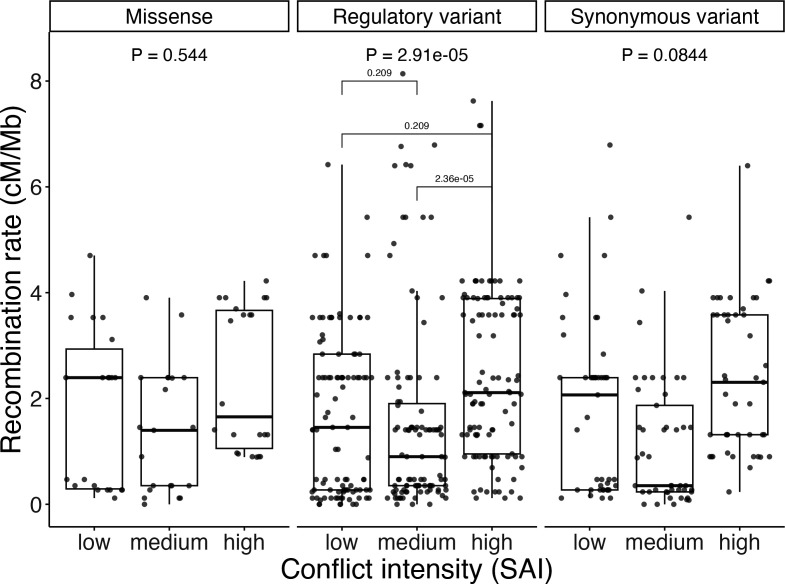
Regulatory variants show a relationship between SAI and recombination rate. Recombination rate is a function of variation in SAI across the three variant types. SAI categories were determined by the tertile of each gene within each variant category. Displayed *p* values are from Brown–Mood test of medians across the three SAI categories.

Focusing only on the fruit-fly candidate genes, we predicted that the three functional categories used in the population genetic analysis (above) may also show a significant association between strength of sexual conflict (SAI) and recombination rate (electronic supplementary material, figure S1), since they each showed the predicted negative relationship between effect size ratio and allele frequency. However, only regulatory variants showed a significant difference in recombination rate across the three categories of conflict intensity (electronic supplementary material, figure S1; Brown–Mood median test, χ^2^ = 26.0, *p* = 2.29 × 10^−6^). Post-hoc testing revealed no significant difference in median between low and medium SAI categories or between low and high SAI categories, but high SAI SNPs showed a higher median recombination rate than the medium SAI category (figure 4).

#### (ii) Gene duplications

**Figure 5.**
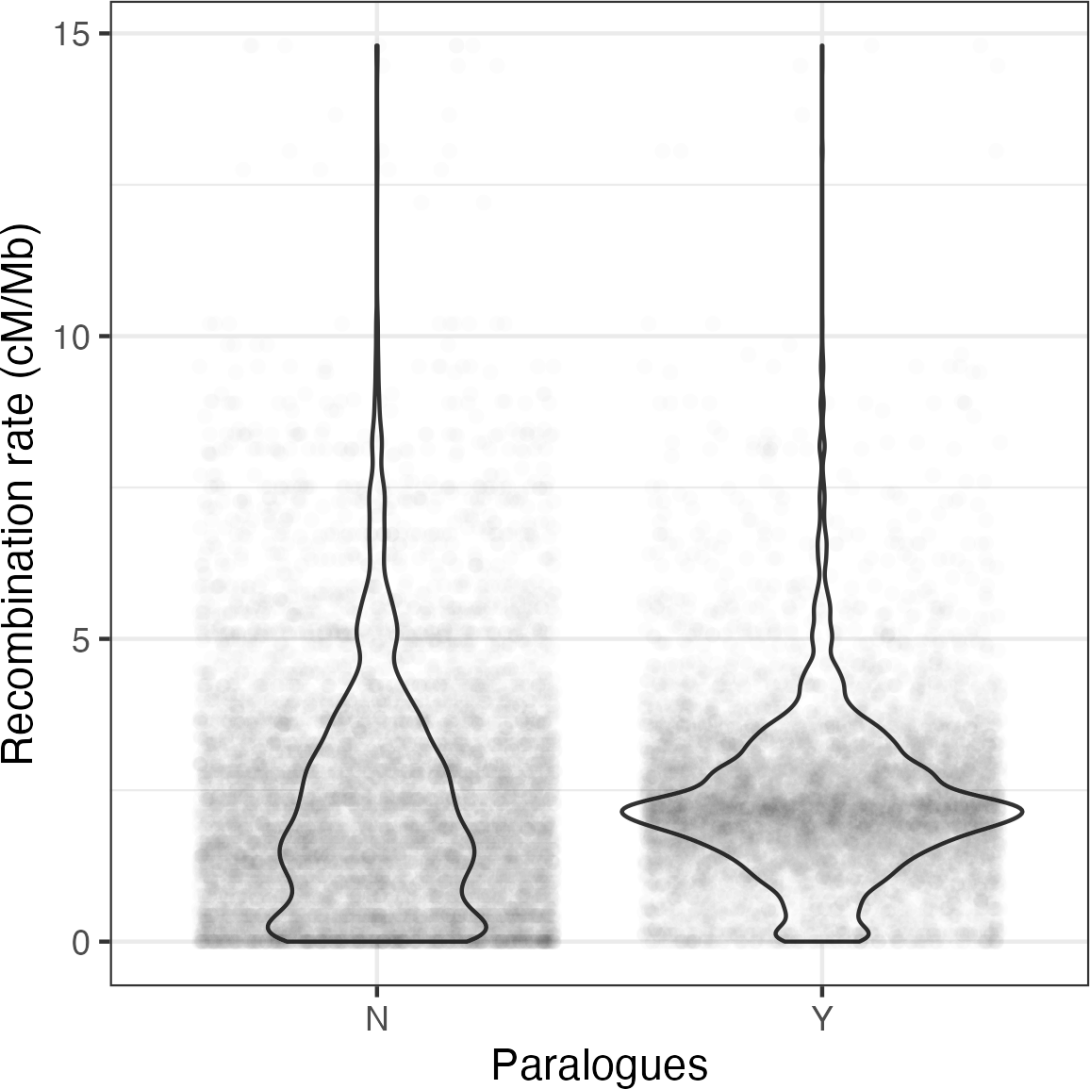
Genome-wide, *Drosophila melanogaster* genes with paralogues occur in regions with higher recombination rates than those without. The recombination rate of genes (cM/Mb) grouped by whether they have paralogues (Y) or not (N).

**Figure 6.**
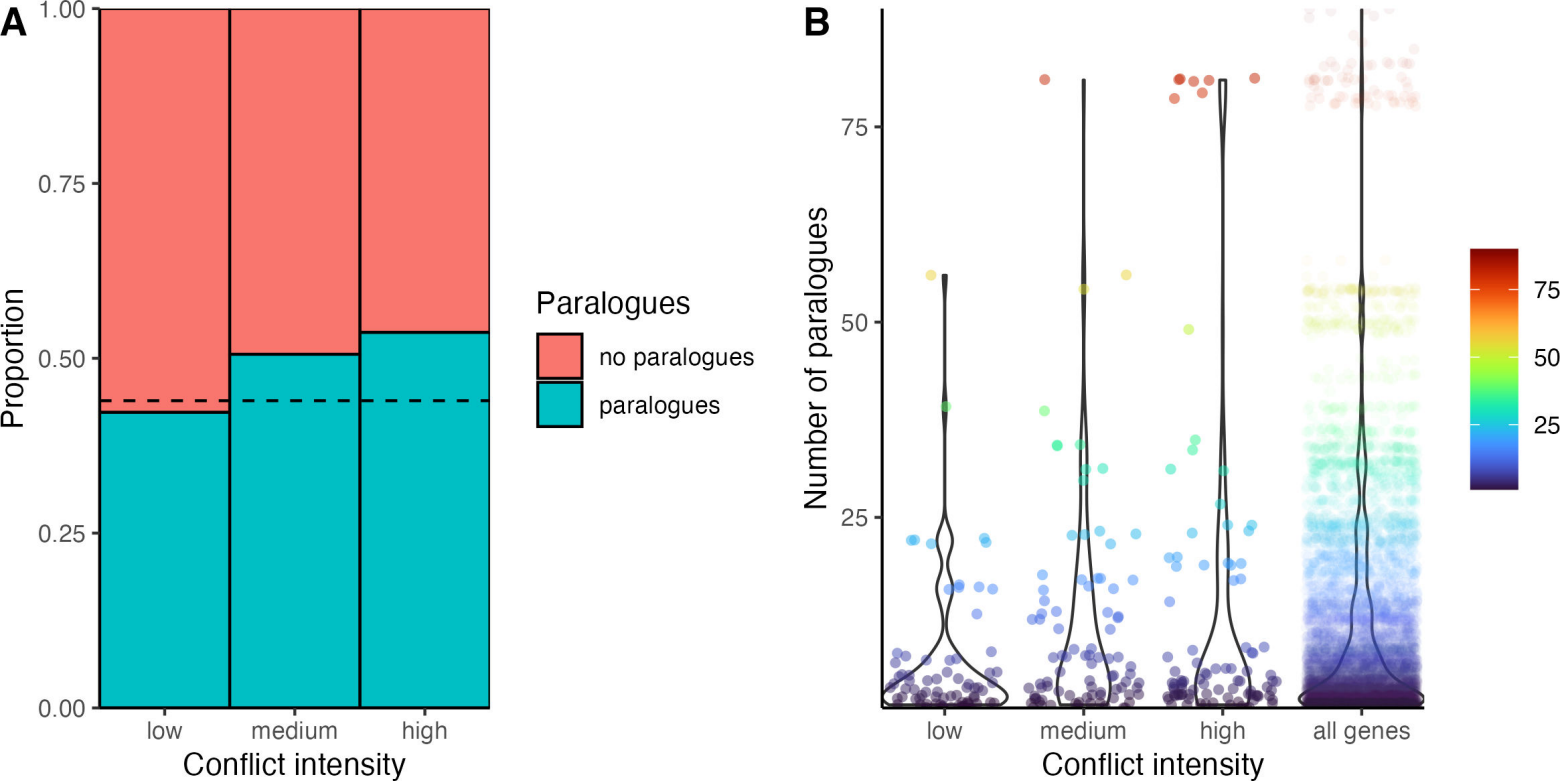
The proportion of genes with and without paralogues and the number of paralogues across the three categories of conflict intensity (SAI). (A) Proportions of genes with paralogues against SAI. Low conflict intensity: χ^2^ = 0.13, d.f. = 1, *p* = 0.719; medium conflict intensity: χ^2^ = 2.82, d.f. = 1, *p* = 0.093; high conflict intensity: *χ*^2^ = 6.33, d.f. = 1, *p* = 0.012. (B) The number of paralogues per gene. For context, the right-most distribution shows data across all fly genes with paralogues. All distributions exclude genes with 0 paralogues. A colour gradient is added to points based on the number of paralogues. Wilcoxon rank sum tests: low-medium *W* = 4102, *p* = 0.0042; low-high *W* = 4242, *p* = 0.0139; medium-high *W* = 4277, *p* = 0.6910.

Generally, *D. melanogaster* genes with paralogues have higher recombination rates than those without paralogues (Wilcoxon rank sum test, *p* < 2.2 × 10^−16^; figure 5). Furthermore, SA candidate genes that experienced the highest intensity of conflict showed a significantly higher proportion of genes with paralogues (0.537) than genes experiencing lower levels of conflict (low: 0.423; medium: 0.506; figure 6A). Finally, genes in the medium and high SAI categories both had higher numbers of paralogues when compared with the low intensity category (figure 6B).

## 4. Discussion

We sought to reduce the large number of candidate SA loci by identifying sets, based on broad functional categories, that are likely to be dominated either by valid SA loci or by false positives. Our rationale was that only valid SA alleles should achieve higher equilibrium frequencies when estimates of their net effects are positive [43]. Across all candidate loci, we were able to confirm this pattern of higher frequencies for alleles with net positive effects (figure 1), which was previously shown for a set of human alleles [41]. We also found the same pattern repeated in each of the three different functional categories the polymorphisms encoded (figure 2). These results suggest SA allelic variation is not limited to protein coding regions but extends to variants that regulate gene expression levels [47], or, as in the case of synonymous variants, by changing the stability of mRNA, which in turn may impact on gene expression levels [54], or through other mechanisms [46]. An alternative explanation is that the association between the allele frequency of non-coding variants and their estimated net effects on male and female fitness is because they are in linkage disequilibrium with SA missense variants. Although still possible for some specific examples, this seems less likely to be a general explanation, as linkage disequilibrium in our population of *D. melanogaster* decays rapidly [42], one of the reasons this species is an ideal model for GWAS [55].

We then explored whether variants in the functional categories that demonstrated support for the population genetic model of SA allele frequencies (missense, synonymous and regulatory) also showed an association between the strength of sexual antagonism and recombination rate. This was based on our hypothesis that there may be an evolutionary advantage to an increase in recombination rate in regions experiencing IASC by promoting gene duplications and conflict resolution. There was support for this idea when looking across all candidate SNPs grouped together, where loci that experience the highest intensity of conflict were associated with higher recombination rates than loci showing the lowest or medium levels of conflict (figure 3*a*). This pattern was largely driven by the regulatory variants, which was the only individual category to show a significant association between strength of sexual antagonism at a locus and recombination rate (figure 4), although there are fewer loci in both the missense and synonymous categories, affecting power. In contrast, this relationship was not found in the set of human loci (figure 3*b*), which at face value is consistent with our hypothesis that meiotic errors at higher recombination rates may be less tolerable for species that produce small numbers of offspring, like humans. However, there is evidence that the genome-wide recombination rate in women is positively related to family size, possibly due to the protective effects of recombination on non-disjunction [56], suggesting the premise for our prediction (iii) does not hold. There were apparently lower levels of conflict in the smaller human set of loci, than found in the *D. melanogaster* dataset (electronic supplementary material, figure S1), which may mean there is either weaker selection for conflict resolution at the human loci. However, this difference could simply be due to either lower power to detect a relationship or other more significant differences between these datasets, as they are based on different proxies for fitness, which makes direct comparisons of conflict intensity problematic. Finally, although not fully resolved, there are distinct differences in the molecular mechanisms underlying recombination and its occurrence in *Drosophila* and mammals, where candidate recombination genes are not conserved [25,57]. Even without invoking an adaptive value for recombination rate, the results from *D. melanogaster* nonetheless suggest that if SA loci coincide with regions with relatively high recombination rates, then stable linkage blocks harbouring SA genetic variation are less likely to be observed, which has been predicted by two-locus models of SA genetic variation [58,59].

We were also able to show that the process of gene duplication genome-wide in *D. melanogaster* may generally be facilitated by higher recombination rates, since genes in families of duplicates were associated with higher recombination rates than singleton genes (figure 5). This result is consistent with many of the other studies that have investigated this relationship (see Introduction), though it does partially run counter to the only other study from *D. melanogaster* [36] which shows a nominally U-shaped relationship between recombination rate and duplicate counts. When looking at SA candidate genes specifically, there was a trend for variants experiencing stronger levels of sexual conflict to show higher proportions of genes with paralogues (figure 6*a*) and greater numbers of paralogues in medium and high levels of conflict (figure 6*b*). Again, these results support the hypothesis that increases in recombination rates may be an adaptive response to IASC in *D. melanogaster* as higher rates of recombination lead to more frequent gene duplication events [60]. This has the potential to facilitate conflict resolution via subfunctionalization of the duplicate genes [7,19,20], should further evolutionary steps follow.

Gene duplicates arising from recombination events remain physically close to the ancestral locus and retain clear homology with the ancestral sequence [23]. Other processes that can cause gene duplications (transposition and retrotransposition) can also result in paralogues being physically close to one another, such as through ectopic recombination between non-allelic homologous retrotransposons [61], but transposons usually result in duplicated copies to a different, more distant locus [62]. Although each of these mechanisms could contribute to the resolution of IASC and would be favoured by selection, a more in-depth analysis that considers genomic distance between gene copies, that is also able to identify the ancestral and duplicate genes (e.g. [63]), would be useful in teasing apart the relative contribution of these processes. Our results also suggest that factors or processes which promote transposition or retrotransposition may also be favoured by selection in the context of resolving IASC, again when balanced against their deleterious effects [61]. Future tests of our hypothesis may also benefit from taking account of interpopulation variation in recombination rate [64], so that data are obtained on recombination rate and SA intensity from closely related or the same populations.

IASC can be seen as a special case of a much broader category of (non-sexual) adaptive conflicts, where genes experience conflicting selection pressures when they perform multiple functions, perhaps at different life-stages, ages or tissues. The result is suboptimal performance across all functions and a population-level genetic load. The escape from adaptive conflict (EAC) model of evolution describes how this conflict can be resolved via gene duplication, since a new duplicate gene can become optimized for a novel function while the ancestral copy is free to become optimized for the ancestral function [65,66]. There are several empirical examples of duplicate genes that have apparently evolved via the EAC model [63,66,67]. For example, duplication of centromeric histone (*Cid*) genes in *Drosophila* resolves an adaptive conflict over functioning during somatic mitosis and combating centromere drive during meiosis [68,69]. Since *D. melanogaster* has only a single *Cid* copy, it may continue to experience this adaptive conflict [69]. Our results showing the potential adaptive response of recombination rate to the presence of IASC suggest there is a testable prediction that selection may also favour increasing rates of recombination as a way of promoting gene duplication and resolution of many different types of adaptive conflict.

## Data Availability

Data files and code can be found at Zenodo [70]. Supplementary material is available online [71].
